# A Spiking Neural Network Model of the Lateral Geniculate Nucleus on the SpiNNaker Machine

**DOI:** 10.3389/fnins.2017.00454

**Published:** 2017-08-09

**Authors:** Basabdatta Sen-Bhattacharya, Teresa Serrano-Gotarredona, Lorinc Balassa, Akash Bhattacharya, Alan B. Stokes, Andrew Rowley, Indar Sugiarto, Steve Furber

**Affiliations:** ^1^Advanced Processor Technologies Group, School of Computer Science, University of Manchester Manchester, United Kingdom; ^2^Instituto de Microelectronica de Sevilla Sevilla, Spain; ^3^Imperial College London London, United Kingdom

**Keywords:** lateral geniculate nucleus, SpiNNaker machine, sPyNNaker, steady state visually evoked potentials, LGN interneurons, entrainment, electronic retina, multi-node models

## Abstract

We present a spiking neural network model of the thalamic Lateral Geniculate Nucleus (LGN) developed on SpiNNaker, which is a state-of-the-art digital neuromorphic hardware built with very-low-power ARM processors. The parallel, event-based data processing in SpiNNaker makes it viable for building massively parallel neuro-computational frameworks. The LGN model has 140 neurons representing a “basic building block” for larger modular architectures. The motivation of this work is to simulate biologically plausible LGN dynamics on SpiNNaker. Synaptic layout of the model is consistent with biology. The model response is validated with existing literature reporting entrainment in steady state visually evoked potentials (SSVEP)—brain oscillations corresponding to periodic visual stimuli recorded via electroencephalography (EEG). Periodic stimulus to the model is provided by: a synthetic spike-train with inter-spike-intervals in the range 10–50 Hz at a resolution of 1 Hz; and spike-train output from a state-of-the-art electronic retina subjected to a light emitting diode flashing at 10, 20, and 40 Hz, simulating real-world visual stimulus to the model. The resolution of simulation is 0.1 ms to ensure solution accuracy for the underlying differential equations defining Izhikevichs neuron model. Under this constraint, 1 s of model simulation time is executed in 10 s real time on SpiNNaker; this is because simulations on SpiNNaker work in real time for time-steps *dt* ⩾ 1 ms. The model output shows entrainment with both sets of input and contains harmonic components of the fundamental frequency. However, suppressing the feed-forward inhibition in the circuit produces subharmonics within the gamma band (>30 Hz) implying a reduced information transmission fidelity. These model predictions agree with recent lumped-parameter computational model-based predictions, using conventional computers. Scalability of the framework is demonstrated by a multi-node architecture consisting of three “nodes,” where each node is the “basic building block” LGN model. This 420 neuron model is tested with synthetic periodic stimulus at 10 Hz to all the nodes. The model output is the average of the outputs from all nodes, and conforms to the above-mentioned predictions of each node. Power consumption for model simulation on SpiNNaker is ≪1 W.

## 1. Introduction

Designing and building brain-inspired computational models has made steady advances following the Nobel-prize winning work by Hodgkin and Huxley (Schwiening, 2012). The biggest challenge in this endeavor has been the inherent serial processing von-Neumann architecture of our computers, making the implementation of parallel information processing complicated and expensive (Markram, 2006; Eliasmith, 2014). Neuromorphic hardware is an emerging trend that has the potential to overcome the constraints of conventional computers in modeling brain structures and functions. Currently, several neuromorphic platforms are under development and available for academic research for example Neurogrid (Benjamin et al., 2014), BrainscaleS (Schemmel et al., 2010), HiAER-IFAT (Yu et al., 2012), SpiNNaker (Furber et al., 2013). Readers may refer to a recent topical review of the current state-of-the-art in neuromorphic computing platforms (Furber, 2016); an introductory-level overview of neuromorphic systems in current times is also discussed in Liu et al. (2016). Our goal in this work is to design and develop a spiking neural network model of the Lateral Geniculate Nucleus (LGN; the thalamic nucleus in the visual pathway) on the novel platform that is SpiNNaker. The motivation is to test the feasibility of simulating biologically plausible behavior on the SpiNNaker machine; this will help in assessing its potential as a “tool” for building large-scale biologically plausible neural networks.

The SpiNNaker machine uses state-of-the-art digital neuromorphic (brain-inspired) hardware based on very-low-power ARM processors and developed under the University of Manchester's SpiNNaker project (Furber et al., 2014). Being biologically inspired, the SpiNNaker machine, also referred to as SpiNNaker, allows brain-like parallel, asynchronous computation, and in real time with time-steps *dt* ⩾ 1 ms. Indeed, one of the primary goals of the SpiNNaker project is to provide users with a massively parallel framework for building neuro-computational tools that run in biologically plausible time-scales. Low power consumption has been an equally important design issue for SpiNNaker, similar to the intelligent energy saving mechanisms in biological networks (Sharp et al., 2012; Stromatias et al., 2013; Knight et al., 2016), and others have demonstrated the significant low power consumption of SpiNNaker compared to supercomputing-clusters when simulating large-scale biological networks. These attributes, combined with an underlying flexible toolchain (Stokes et al., 2017), make SpiNNaker a potentially “fertile” base for emulating bespoke neuro-computational models applied to both clinical neuroscience (for example emulating higher level brain dynamics Sen-Bhattacharya et al., 2014) as well as intelligent machines (for example cognitive robotics Adams et al., 2014).

The visual pathway has drawn considerable research interest from neuroscientists over several decades (Pasternak et al., 2003) perhaps due to the “ease of access” to the retina and the optic nerve, allowing detailed physiological studies. This has resulted in extensive information being available on the organizational and functional mechanisms of vision (Wurtz and Kandel, 2000). Thus, it has emerged that the LGN plays a fundamental role in enhancing visual attention and cognition; this in addition to what has been traditionally thought to be its primary role of relaying retinal information to the visual cortex (Sherman and Guillery, 2001). The computational model of LGN in this work consists of three cell populations as in biology viz. thalamo-cortical relay (TCR), thalamic interneurons (IN) and the thalamic reticular nucleus (TRN). The basic unit of the network is an Izhikevich's model of a spiking neuron (Izhikevich and Edelman, 2008), a popular framework for spiking neuron modeling owing to its versatility and yet, low computational requirements. The structural layout of the model is based on physiological data obtained from the dorsal-LGN (LGNd) of cats and rats (similar to previous works, Sen-Bhattacharya et al., 2016). All LGN cells are known to fire in two modes, viz. tonic and burst, depending on the functionality of the circuit (Sherman, 2001); tonic firing is often associated with the state of attention and information processing (Weyand et al., 2001). For the purposes of this work, we follow a “simple” assumption that all cells of the LGN fire in the tonic mode when processing sensory information received from the retinal spiking neurons.

The first objective toward our goal in this work is to make a feasibility study of simulating steady state visually evoked potentials (SSVEP)—brain oscillations recorded in electroencephalogram (EEG) corresponding to periodic visual stimuli—using the model. Our approach is justified by experimental research showing a strong correlation between local field potentials (LFP) recorded from the LGN and EEG recorded from the occipital scalp electrode i.e., the location of the visual cortex (da Silva et al., 1973; Crunelli et al., 2006). Moreover, after several decades of experimental research on isolated thalamic slices of mammals and rodents, it is now well understood that the LGN plays a fundamental role in generating and sustaining cortical oscillations observed via EEG (Timofeev and Steriade, 1996). SSVEP have been used for several decades now to study visual perception (Norcia et al., 2015). Indeed, the paradigm continues to grow in popularity for its relatively easy “frequency tagging” characteristics, which can have significant applications in the area of clinical neuroscience as well as in Brain Computer Interfaces (Vialatte et al., 2010; Diez et al., 2011; Guger et al., 2012). Such frequency tagging, in turn, is facilitated by the inherently stable spectrum of SSVEP signals and their high signal to noise ratio. While the underlying neural mechanisms of the harmonics and subharmonics in the SSVEP signals are as yet unclear, there is a general understanding that the non-linearity of the visual system plays a major role (Hermann, 2001). A recent computational model-based study that was validated with experimental data further emphasized the human visual system non-linearity as a causal factor in SSVEP harmonics (Labecki et al., 2016). The authors of another recent experimental work have argued that entrainment of brain signals by visual rhythmic (periodic) stimulation underlies the “origin of SSVEP” (Notbohm et al., 2016). In this work, we validate the power spectra of the LGN model output with that corresponding to SSVEP reported in Hermann (2001) and Labecki et al. (2016).

A recent report by Hirsch et al. (2015) emphasizes the role of feed-forward inhibition in the retino-geniculate pathway, viz. from the retina to the TCR cells via the IN, in maximizing the information transmitted to the cortex by the TCR population. This is not surprising as the IN population is known to receive around 47% of its synaptic afferents by the retinal spiking neurons compared to only 7.1% from the TCR cells. Interestingly, the rat thalamus is found to be devoid of IN cells, the only exception being the LGN; this further emphasizes the significance of IN in the visual pathway of both mammals and rodents. However, computational model-based research on thalamo-cortical dynamics of health and disease largely ignore the feed-forward inhibition by the IN. In contrast, the feed-forward and -back connections between the TCR and TRN have been studied extensively by both neuroscientists and modellers toward understanding EEG and LFP in health (Huntsman et al., 1996) as well as in neurological disorders, for example in epilepsy (Wang et al., 2014). A recent mesoscopic-scale neural-mass model based study has reported a causality of IN connectivity with LGN dynamics (Sen-Bhattacharya et al., 2016). Motivated by this model-based observations, the second objective in this work is to test the LGN model on SpiNNaker for causality of the IN pathway on the simulated SSVEP power spectra characteristics.

In summary, the aim of the work is three-fold: first, to design and develop a spiking neural network model of the LGN on the novel platform that is SpiNNaker; second, to simulate SSVEP-like signals in the model; third, to test the causality of feed-forward inhibition in the retino-geniculate circuit on model dynamics. It is worth mentioning here that the current work is an initial attempt to simulate a biologically plausible LGN spiking neural network on the SpiNNaker machine. Thus, benchmarking performance evaluation of SpiNNaker with other available neuromorphic platforms when simulating the LGN model is outside the scope and objectives of our current work. Rather, the emphasis is on testing the SpiNNaker platform as a viable device to simulate biologically plausible neural network behavior. Furthermore, such instances of neural model simulation also contribute toward enhancing the development of the sPyNNaker toolchain by providing crucial feedback from time to time for example on fixing bugs.

In Section 2, we present the methods of modeling the LGN, followed by some background information on the two unique hardware platforms used in this work: the SpiNNaker machine and the electronic retina. In Section 3, we present the results from this study as well as specify the simulation, data collection and power evaluation methods. A discussion on the results as well as on future works that can build on the framework presented herewith is provided in Section 4. We conclude the paper in Section 5.

## 2. Materials and methods

In Section 2.1, we present an overview of the LGN model and its biologically informed synaptic layout. In Section 2.2, we present the parameterization of the spiking neural network in the model. The SpiNNaker toolchain, sPyNNaker, forms the backbone of all neuronal model implementations on the SpiNNaker machine and is discussed in Section 2.3. An overview of the electronic retina is presented in Section 2.4.

### 2.1. Synaptic layout of the lateral geniculate nucleus model

**Figure 1:**
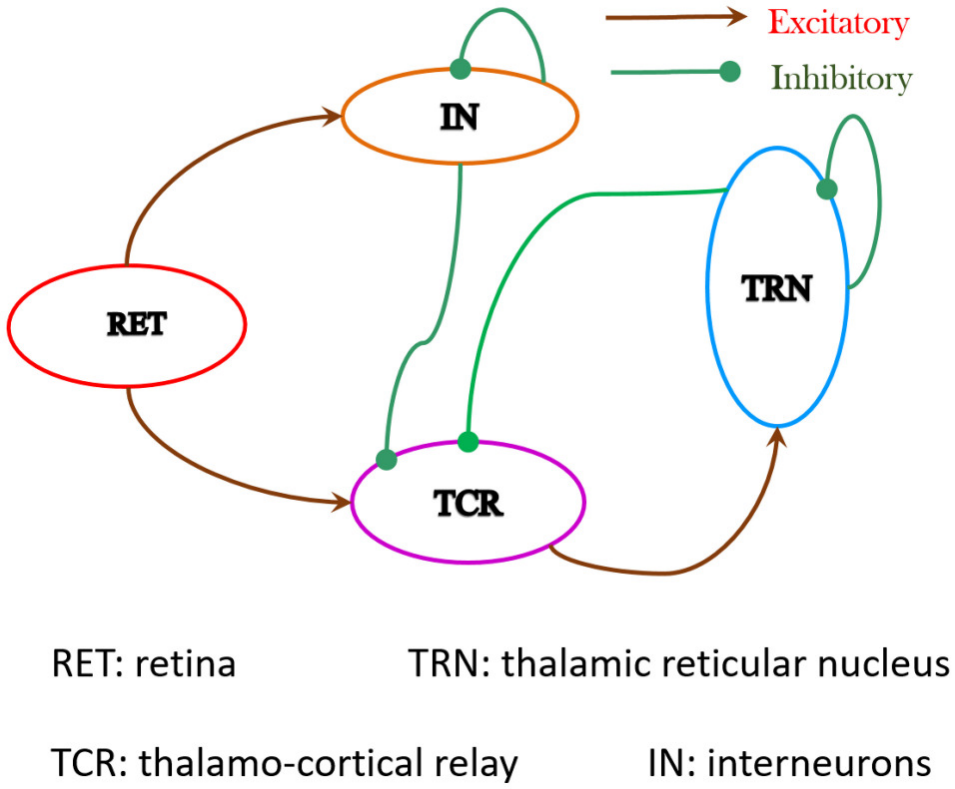
The synaptic layout of the model is based on experimental data obtained from the dorsal Lateral Geniculate Nucleus (LGNd) of mammals and rodents. Both TCR and IN cells of the LGN receive excitatory inputs from the retinal spiking neurons. The IN cells make inhibitory synapses on themselves as well as on the TCR cells. Information on the synaptic pathway from the TCR to IN is ambiguous in literature and is ignored here. The TCR cells make excitatory synapses on the TRN cells. The TRN cells makes inhibitory synapse on the TCR cells as well as on themselves. The synaptic connectivities in the network are sparse and the probabilities of connectivity *p*_*conn*_ between pre-synaptic and post-synaptic populations in the model are mentioned in Table 1.

The synaptic layout of the LGN model in this work is shown in Figure 1. Sensory information from the retina is carried by the ganglion cell axons, which constitute the optic nerve, to the LGN; the ganglion cells make excitatory synapses on both the TCR and IN cells. The axons of the TCR cells are the main carriers of the sensory information to the visual cortex (to Layer 4 primarily); the visual cortex (Layers 5 and 6 primarily) is known to send vital feedback signals to both TCR and IN cells of the LGN. The TRN is a thin sheet of neuronal tissue that surrounds the dorsal thalamus. All information carrying pathways from the TCR to the visual cortex have major branches that make excitatory synapses on to the TRN cells; similarly, all feedback from visual cortex to the TCR and IN cells is also communicated to the TRN via axonal branches. Thus, it is not surprising that the TRN is located strategically, which allows it to “monitor” all communication between the LGN and the visual cortex. In this work, however, our objective is to understand the dynamics of the de-decorticated (disconnected from the cortex) LGN dynamics; thus we have not looked into thalamo-cortical or cortico-thalamic connections. This is similar to experimental studies on LGN slices *in vitro* (Bal et al., 1995) that paved the way for a deeper understanding of the thalamo-cortico-thalamic dynamics, which has been referred to as the “Rosetta stone” of neurological disorders (McCormick, 1999).

The LGN comprises around 70–80% TCR cells and 20–25% IN cells, suggesting the ratio of TCR to IN as ≈4:1 (Sherman and Guillery, 2001). There is no available corresponding data on the TRN to the best of our knowledge, and is arbitrarily set in this model as twice the number of IN cells. The proportion of cells in the TCR, IN and TRN populations is set as 8:2:4. The LGN model is populated with a total of 140 neurons, and is intended to serve as a “basic building block” for scaled-up model versions toward building massively parallel frameworks on SpiNNaker. Each neuro-computational “unit” of the model is the Izhikevich's spiking neuron model, defined in Equations (1)–(3) (Section 2.2).

**Table 1:** Normalized base parameter values of synaptic projection from the pre-synaptic population to the post-synaptic population expressed as a probability *p*_*conn*_ ∈ (0, 1).

**Post-synaptic** → **Pre-synaptic** ↓	**TCR**	**IN**	**TRN**
TCR	X	X	0.35
IN	0.232	0.236	X
TRN	0.077	X	0.20
RET	0.071	0.474	X

*The values are derived from experimental data on LGN of mammals and rodents (Horn et al., 2000; Jones, 2007) (see Section 2.1 for a brief overview). To the best of our knowledge, specific data distinguishing the TRN and IN afferent terminals on the TCR population are yet to be reported. Thus, we set each of these parameters to a fraction $x\in \left\{\frac{3}{4},\frac{1}{4}\right\}$ of 0.309, such that the combined strength of inhibitory input to the TCR from the TRN and IN is 30.9% as reported in literature. All “X” indicate a lack of biological evidence for synaptic connectivity in the specific pathway*.

Detailed physiological data reporting the synaptic afferents (“fan-in”) and efferents (“fan-out”) for each LGN cell types is scarce, understandably due to the expensive and complicated overheads of experimental research, and the dependence on advancements of recording equipments. Even for the few existing studies, there is a significant deviation in reported data depending on the species and animal being studied, as well as in the experimental methods for data collection. In prior works on parameterizing LGN models (Sen-Bhattacharya et al., 2011b, 2016), we have followed the most recent available physiological data reported by Horn et al. (2000), which is subsequently confirmed in Sherman and Guillery (2001) and Jones (2007). A brief account of the intra-LGN connectivity is provided below and the related parameters are mentioned in Table 1.

Data from the cat LGNd suggest that the TCR receive ≈7.1% of their inputs from the retinal ganglion cells, while ≈30.9% of their inputs are from inhibitory sources viz. IN and TRN. However, and to the best of our knowledge, there is no data available that distinguishes between the afferent synaptic terminals from the IN and TRN due to similarity in their physical shapes and sizes. Thus, we set each of these parameters to a fraction $x\in \left\{\frac{3}{4},\frac{1}{4}\right\}$ of the maximum value 0.309 such that the total strength of inhibitory input to the TCR is 30.9% as reported in literature. Furthermore, and aligned with the aims of our work as outlined in Section 1, the feed-forward inhibitory pathway connectivity is dominant in the “base” (reference) model state. (In Section 3, we study the effects of a diminished role of the feed-forward inhibition from IN on sensory information processing by the LGN). The remaining ≈62% of the TCR afferents are from the cortex as well as other sub-cortical sources, and are outside the scope of the current work.

As reported in Horn et al. (2000), the IN cells receive around 47.4% synapses from the retinal spiking neurons, 23.6% from other inhibitory sources including themselves, while 29% synapses are from the cortex. It may be noted that the IN circuitry in the LGN has a unique triadic spiking arrangement (Sherman, 2004) consisting of dendrites that are not only post-synaptic to the retinal cells but are also pre-synaptic to the TCR cells as well as to themselves (dendro-dendritic synapses). These are referred to as the F2 terminals of the IN while the usual pre-synaptic axonal terminals are referred to as F1. However, the exact distribution of F1 and F2 terminals is not yet available from physiological studies; thus the above-mentioned figures for synaptic afferents to the IN refer to the combined numbers of both types of terminals. Also worth mentioning here is that experimental observations of IN cell dynamics do mention an excitatory feed from the TCR to these cells (Crunelli et al., 1988; Zhu et al., 1999; Lörincz et al., 2008), however, these were speculations based on cell behavior as opposed to cell physiology. On the other hand, experimental studies on IN cell physiology suggest two specific cell types in the LGN (Cox et al., 2003): the intra-layer IN cells that do not receive any afferents from the TCR cells; the inter-layer IN cells that do receive inhibitory feedback from the TCR cells, and are often thought to be “stray” cells of the TRN. In the present work, we consider the intra-layer IN cell population only; thus, as in our previous work (Sen-Bhattacharya et al., 2011b), the IN population do not receive any synaptic afferents from the TCR.

Connectivity for the TRN population follows the data reported in Jones (2007) and is obtained from the rat LGNd and visual cortex. Both thalamocortical and corticothalamic synapses on the TRN sector are excitatory in nature and constitute ≈30–40% and ≈50% respectively of the total synapses; the remaining up to 25% of the synapses are from other inhibitory sources including neighboring intra-population cells. In our model, we maintain the excitatory afferents from the TCR as 35% (average of the reported figure), and self inhibitory connectivity within the TRN population (consistent with previous works Sen-Bhattacharya et al., 2016) as 20%.

It may be noted that the above-mentioned physiological data are based on the number of synaptic vesicles observed through electronic microscopy on dendritic boutons that are located closer to the soma (as opposed to distal dendritic locations) (Horn et al., 2000). Furthermore the figures are normalized (percentage), thus providing a relative estimate of the maximum possible fan-in per “unit neuronal representation” in a network. In this model, we set this normalized figure as the probability of connectivity from one neuronal population to the other (discussed further in the following section). Thus, all figures mentioned in the table are expressed as a probability *p*_*conn*_ ∈ (0, 1).

### 2.2. Parameterizing the LGN network

Each Izhikevich neuron model in this work is as defined in Equations (1)–(3) (Izhikevich, 2003) and parameterized for generating tonic spiking pattern corresponding to a cognitive brain state:

(1) $$\begin{array}{r} \frac{dv\left(t\right)}{dt}=0.04v^{2}\left(t\right)+5v\left(t\right)+140-u\left(t\right)+I_{psc}\left(t\right)+I_{dc} \end{array}$$

(2) $$\begin{array}{r} \frac{du\left(t\right)}{dt}=a\left(bv\left(t\right)-u\left(t\right)\right) \end{array}$$

(3) $$\begin{array}{r} \text{If }v\left(t\right)>30,\text{then }v\left(t\right)\leftarrow c;u\left(t\right)\leftarrow u\left(t\right)+d \end{array}$$

where, *a, b, c, d* are parameters that define the dynamic behavior of the model and can be tuned to obtain various spiking patterns as observed in biology (Izhikevich, 2004); *v*(*t*) is the membrane potential and *u*(*t*) is a membrane recovery variable (Galbraith, 2011); *I*_*psc*_(*t*) is the post-synaptic current due to all extrinsic spike inputs to the model; *I*_*dc*_ is a constant bias current stimulus to the model that forms part of the neuron model definition on sPyNNaker. While the original works by Izhikevich (2003) preferred not to provide units for the parameters a, b, c, d, and u, subsequent works have indeed assigned units to these parameters (Elibol and Sengör, 2015); we preferred to follow the original work. The base parameter values of Izhikevich's model neurons used in the LGN model are mentioned in Table 2.

**Table 2:** The base parameter values for Izhikevich's neuron model used in the LGN network to produce spiking dynamics shown in Figure 2.

**LGN cells**	**Spiking pattern**	**a**	**b**	**c**	**d**	***v*_*i*_ (mV)**	***u*_*i*_**
TCR	*RS*	0.02	0.2	−65	6	−65	−13
IN	*FS*	0.1	0.2	−65	6	−70	−14
TRN	*RS*	0.02	0.2	−65	6	−75	−15

*The initial values of the variables v and u in Equations (1) and (2) are represented by v_i_ and u_i_, respectively. RS, Regular Spiking; FS, Fast Spiking*.

It is believed that when firing in tonic mode, the TCR cells display a Regular Spiking(RS) behavior (Izhikevich, 2003). The spiking behavior of the TRN cells is also thought to be similar to the TCR cells (Halassa and Acsady, 2016). Thus, in this work, the Izhikevich's model neurons for simulating each TCR and TRN neuron are parameterized to fire in the RS mode. On the other hand, the IN cells are reported to display a Fast Spiking (FS) behavior (Izhikevich, 2003) and are modeled accordingly in this work.

**Figure 2:**
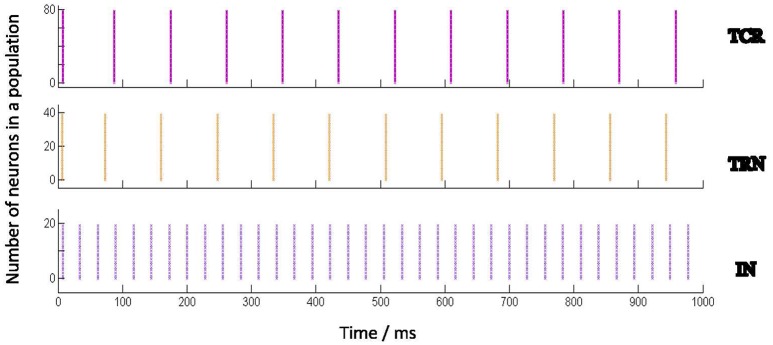
Behavior of the Izhikevich model neurons when acting independently (i.e., not connected to one another) and stimulated by a constant input current (*I*_*dc*_). Each Izhikevich model neuron simulating a TCR and TRN cell is parameterized to fire in a Regular Spiking (RS) mode corresponding to *I*_*dc*_, while each IN cell is parameterized to fire in a Fast Spiking (FS) mode. The corresponding parameters are mentioned in Table 2. The case for *I*_*dc*_ *= 5 nA* is shown here. The Implementation of the Izhikevich neuronal models are as defined in PyNN (Davison et al., 2009) and mapped on SpiNNaker by the underlying toolchain sPyNNaker version 3.0.0 (Stokes et al., 2017). *I*_*dc*_ forms part of the Izhikevich neuron definition on sPyNNaker, therefore acting as current stimulus to each neuron model from the start of simulation time. The raster plots show that all neurons in a certain population, when acting independently, respond similarly to a stimulus in terms of spike times and frequency.

Figure 2 shows the spiking pattern of each cell population corresponding to a constant value of *I*_*dc*_ (nA). There are no other extrinsic inputs to the circuit (i.e., *I*_*psc*_ = 0), and all network connectivities are removed (i.e., *p*_*conn*_ = 0). Thus, all the neurons in the model are firing independently of one another, and are just dependent on *I*_*dc*_. Furthermore, as *I*_*dc*_ forms part of the neuron model definition on sPyNNaker, it is present as a stimulus to all neurons in the model from start of the simulation. The raster plots in Figure 2 demonstrate that all neurons in a certain population respond similarly (in terms of spike time and frequency) to the same stimulus. The respective frequencies of spiking increase for all populations with increasing values of *I*_*dc*_.

**Figure 3:**
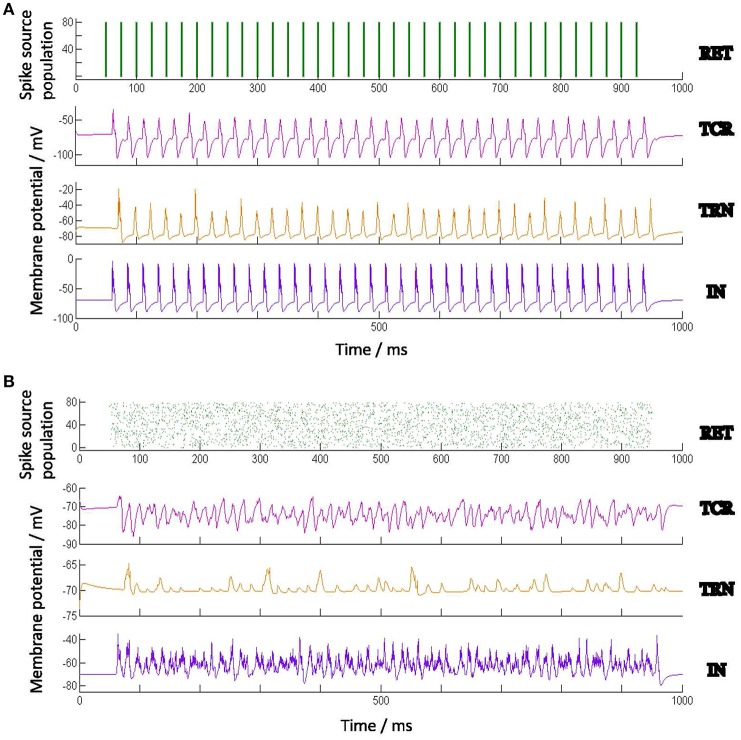
Average population membrane potential with **(A)** periodic spike-train, and **(B)** Poisson spike-train input at 40 Hz. All neuronal and connectivity parameters are as in Tables 1–3.

Next, the cell populations are connected as a network as shown in Figure 1. The constant current bias *I*_*dc*_ for all cell populations are now assigned a value of 0; thus the total current in the model is only due to *I*_*psc*_ that is initiated by retinal spiking inputs simulated by both periodic and aperiodic spike trains, the latter following a Poisson distribution. The model inputs and corresponding response patterns are shown in Figures 3A,B. Each synaptic connectivity in the model network consists of three attributes: (a) The weight *w*_*syn*_ of the synapse—this emulates a synaptic weight assigned to the post-synaptic membrane current *I*_*syn*_ and is defined thus:

(4) $$\begin{array}{l} I_{syn}\left(t\right)=w_{syn}e^{-t/\tau_{syn}},syn\in \left\{e,i\right\}, \end{array}$$

where “*e*” and “*i*” denote excitatory and inhibitory synapses respectively, and τ_*syn*_ is the time constant of the synapse. Equation (4) is solved separately to the Equations (1)–(3) at every simulation time-step of the model; all equations are solved in SpiNNaker. The values for the parameter *w*_*syn*_ are adjusted to be above a minimum threshold required to effect action potential in the post synaptic cells in the network and are mentioned in Table 3. The parameters τ_*syn*_ are mentioned in the legend of Table 3 and are as in Roth and van Rossum (2009), which in turn are based on physiology data from thalamo-cortical circuit. (b) The delay of the synaptic connection *d*_*conn*_—this represents the latency of a pre-synaptic cell spike in reaching the post-synaptic cell. The delay parameters in this model are set arbitrarily in arithmetic progression to reflect the overall distance between the pre- and post-synaptic cells; thus, the delays for the self inhibitory projections in the TRN and IN are the minimum, while those for the external spike source input projections to the LGN cells are the maximum. The parameter values are provided in Table 3. (c) The probability of the synaptic connection *p*_*conn*_ ∈ (0, 1)—this parameter, discussed in Section 2.2, takes into account the sparsity of the biological networks and defines the total “fan-in” from the pre-synaptic population to the post-synaptic population. Parameter values are mentioned in Table 1.

**Table 3:** The synaptic weights *w*_*syn*_ in the model are parameterized so as to be above the minimum threshold for spiking corresponding to a Poisson train input, the corresponding thresholds for periodic spike-train inputs being comparatively lower.

**Post-synaptic** → **Pre-synaptic** ↓	**TCR**	**IN**	**TRN**
TCR	X	X	*w*_*syn*_ = 3 (nA)
			*d*_*conn*_ = 8 (ms)
IN	*w*_*syn*_ = 8 (nA)	*w*_*syn*_ = 2 (nA)	X
	*d*_*conn*_ = 6 (ms)	*d*_*conn*_ = 4 (ms)	
TRN	*w*_*syn*_ = 2 (nA)	X	*w*_*syn*_ = 2 (nA)
	*d*_*conn*_ = 8 (ms)		*d*_*conn*_ = 4 (ms)
RET	*w*_*syn*_ = 5 (nA)	*w*_*syn*_ = 4 (nA)	X
	*d*_*conn*_ = 10 (ms)	*d*_*conn*_ = 10 (ms)	

*The delay parameters d_conn_ are arbitrarily set as progressively diminishing so as to indicate maximum delay (10 ms) for inter-population projections and minimum delay (4 ms) for recurrent projections. The post-synaptic projections follow an exponential decay with time constant τ_e_ = 1.7 ms for excitatory synapses, and τ_i_ = 2.5 ms for inhibitory synapses (Roth and van Rossum, 2009)*.

### 2.3. Overview of SpiNNaker toolchain interface

The SpiNNaker (short for “Spiking Neural Network architecture”) machine consists of a large number of very-low-power ARM processing units, coupled together by a novel low-powered network architecture designed to send small messages (i.e., neural spikes) across the system to multiple destinations simultaneously, using a SpiNNaker-specific multicast protocol to ensure efficiency. Each chip of the machine has up to 18 ARM 968 cores, each of which operates at 200 MHz, and has access to 32 K of instruction memory, 64 K of data memory, and a shared SDRAM of 128 MB. Though these numbers are relatively small by modern computing standards, the machine is highly extendable, with boards containing 48-chips each (approaching 1,000 cores on a board) being coupled together to yield a machine consisting of up to a million such cores.

Users of the SpiNNaker software such as those from the computational neuroscience community are often not in a position to write neural code that is optimized to execute efficiently on the SpiNNaker platform. The SpiNNaker software, sPyNNaker (Stokes et al., 2017), allows these users to define neural networks using the PyNN description language (Davison et al., 2009), which is then compiled into appropriately sized units of computation and communication, which in turn are placed onto the machine in such a fashion that ensures that real time constraints are met and that all packets are delivered during the simulation. The sPyNNaker software then executes the simulation for a pre-determined time period and extracts the results from the SpiNNaker machine to the host for post processing. One obvious advantage of this is that a certain simulation running in “X” seconds on SpiNNaker is guaranteed to complete execution in that time for every run.

The sPyNNaker software release 3.0.0 (Stokes et al., 2017), used to simulate the model in this paper, contains a fair variety of neuron model implementations including the widely used Leaky Integrate and Fire model and the Izhikevich neuron model. As indicated in Section 1, the LGN circuit in this work is implemented using the Izhikevich's neuron model [Equations (1)–(3) in Section 2.3] with exponentially decaying current-based synapses (specified in PyNN as “IZK-curr-exp”) (Hopkins and Furber, 2015). The differential equations of IZK-curr-exp are solved with a 2nd order Runge-Kutta method.

Readers may note that the SpiNNaker hardware is designed inherently to simulate in real time for time-steps ⩾1 ms. Recently, the sPyNNaker toolchain was modified to support smaller time-step resolutions; however the current implementation of the software can result in the simulation not maintaining synchronization when run in real time. Thus, a technique is implemented by default on the configuration file (spynnaker.cfg) whereby an user application requiring time-step <1 ms is automatically set to run at a time that is scaled up by the inverse of the time-step. For example, if the time-step of a certain simulation is 0.1 ms and the total simulation duration is 1 s, then by default the simulation will run in 10 s real time. However, the users can override the default in the configuration file according to their application requirements.

With regards to accessibility by wider audience, we would like to note that the SpiNNaker machine comprising over half-a-million cores has now been made available via the Neuromorphic platform within the Human Brain Project Collaboratory (Muller, 2016). This allows users to submit jobs to the machine where they are managed using a custom batch processing system; users are notified via email when results are available for collection. The facility is a step forward in facilitating easy access to the SpiNNaker computing platform.

### 2.4. The electronic retina

**Figure 4:**
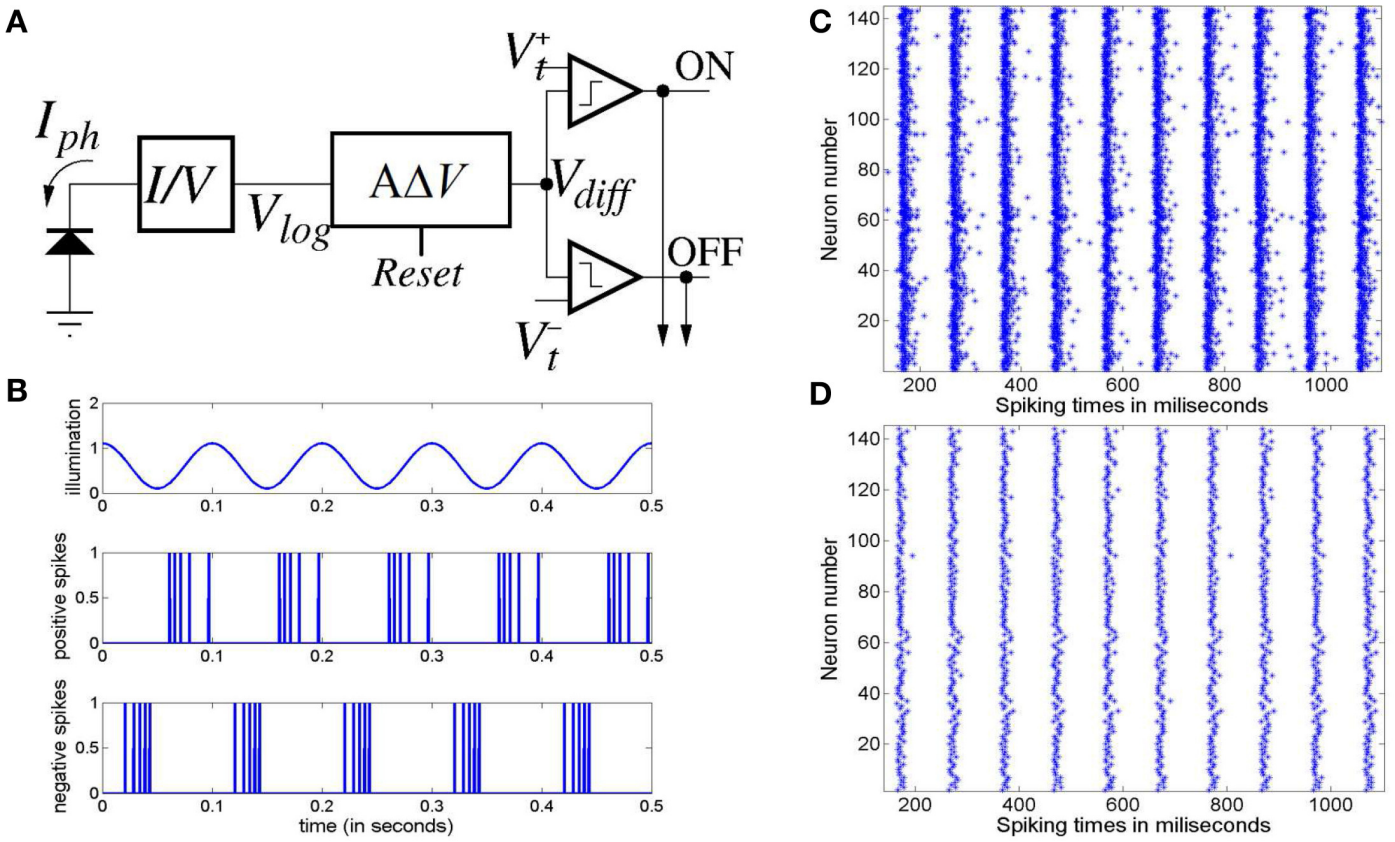
**(A)** Conceptual block diagram of a Dynamic Vision Sensor (DVS) pixel. **(B)** Illustration of the spikes generated by a DVS pixel with a sinusoidal illumination. Upper trace represents the pixel illumination versus time. Middle trace represents the positive spikes generated by the pixel along time. Lower trace represents the negative spike timings. **(C)** Raster plot of the positive spikes generated by 12 × 12 pixels of the electronic retina subjected to a 10 Hz flashing LED during 1 s, and **(D)** raster plot of the first positive spike generated by the same 12 × 12 pixels of the electronic retina after each illumination positive transition during the same 1 s.

The electronic retina used in this work is a Dynamic Vision Sensor (DVS) (Serrano-Gotarredona and Linares-Barranco, 2013), a novel bio-inspired technology that emulates the spiking behavior of the retinal ganglion cells (Lichtsteiner et al., 2008; Lenero-Bardallo et al., 2011; Posch et al., 2011, 2014). Each pixel in a DVS senses the reality in a continuous way and autonomously generates output spikes whenever it detects temporal variations in the illumination impinging on it; the rate of output spikes is proportional to the relative change in the illumination. If the relative change in the illumination impinging on the pixel increases over a given threshold value, the pixel will generate a positive output spike; conversely, when the relative change of the pixel illumination decreases below a given threshold the pixel will generate a negative output spike. Figure 4A illustrates the underlying circuit of a DVS pixel:

The illumination impinging on the pixel is detected by a photo-diode which converts it to a current *I*_*ph*_. A logarithmic block transforms the photo-current into a voltage *V*_*log*_ such that *V*_*log*_ = *K*·*Log*(*I*_*ph*_), where *K* is some transformation constant; the voltage *V*_*log*_ is the input to a differentiator block with a “Reset” input signal. Whenever the Reset signal is activated, voltage *V*_*diff*_ is set to some reference value *V*_*ref*_, where $V_{t}^{-}<V_{ref}<V_{t}^{+}$, and $V_{t}^{+/-}$ are two comparison threshold voltages. When the Reset signal is deactivated, the output of the differentiator follows the variation of the input voltage *V*_*log*_ through the following relation:

(5) $$\begin{array}{r} \Delta V_{diff}=A\cdot \Delta V_{log}=A\cdot K\cdot \frac{\Delta I_{ph}}{I_{ph}} \end{array}$$

Voltage *V*_*diff*_ is compared with the two thresholds voltages. When $V_{diff}>V_{t}^{+}$, it implies that the variation of *V*_*diff*_ since the last reset verifies $\Delta V_{diff}>V_{t}^{+}-V_{ref}$. Thus a positive relative increase in the current has taken place since last reset verifying $\frac{\Delta I_{ph}}{I_{ph}}>\frac{\left(V_{t}^{+}-V_{ref}\right)}{A\cdot K}$. In that case, the positive comparator would trigger a positive (“ON”) output spike and voltage *V*_*diff*_ will self-reset to value *V*_*ref*_. In a similar way, when voltage *V*_*diff*_ goes below threshold $V_{t}^{-}$, it means that a negative relative variation in the current verifying that $\frac{\Delta I_{ph}}{I_{ph}}<\frac{\left(V_{t}^{-}-V_{ref}\right)}{A\cdot K}$ has taken place since last pixel reset, so that the pixel would generate a negative (“OFF”) output spike and voltage *V*_*diff*_ will reset to *V*_*ref*_.

Figure 4B illustrates the behavior of the DVS pixel under a sinusoidal illumination varying with a frequency of 10 Hz. The upper trace represents the pixel illumination along time. The middle trace illustrates the positive spikes generated versus time. As can be observed, when the illumination increases the pixel generates positive spikes and the spikes are denser when the relative change in the illumination is higher. In a similar way, the lower trace illustrates the negative spikes generated by the pixel when the illumination decreases.

## 3. Results

In Section 3.1, we present the experimental set-up for data acquisition from the electronic retina. Methods for model simulation, data processing and visualization are presented in Section 3.2. The observation from the model and results are presented in Sections 3.3–3.6. An estimation of the power consumed by simulation of a single instance of the model is presented in Section 3.7.

### 3.1. Experimental set-up of electronic retina

To emulate the behavior of the model under a real periodic visual stimulus, we have recorded the spikes generated by the electronic retina when placed in front of an LED that is driven by a periodic signal, thus producing a flashing light. This is similar to experimental studies of SSVEP on humans and animals. For the purposes of this work, the electronic retinal spike-train corresponding to periodic LED flashing at 10, 20, and 40 Hz are recorded. As illustrated in Figure 4B, each pixel of the retina produces a series of positive and negative spikes with every rising (ON) and falling (OFF) edge of the flashing light respectively.

Figure 4C illustrates the raster plot of the spikes generated by 12 × 12 pixels of the retina stimulated by the LED flashing at 10 Hz recorded during 1 s. For brevity in this work, we assume ON-center receptive fields for all retinal cells, and just consider the positive spikes in response to the switching ON of the LED. Furthermore, previous research indicates that around 80% of the input information can be recovered from “first spikes” emitted by retinal ganglion cells observing a static monochrome image (Sen-Bhattacharya, 2008). Along these lines, we consider only the first spike generated by each pixel after the switching ON of the diode; the corresponding raster plot is shown in Figure 4D. A similar procedure was followed for the spikes recorded at 20 and 40 Hz. The spike-train output of the retina recordings are provided as input to the model; all simulation methods are the same as with the synthetic model input, which is elucidated in the following section.

### 3.2. Simulation and data processing methods

Total simulation time of the model is set to 6 s. The time resolution of simulation is set to 0.1 ms for ensuring solution accuracy of Izhikevich's neuron models defined in Equations (1)–(3). As elucidated in Section 2.3, our total simulation duration is thus 60 s in real time, where 0.1 ms simulation time runs on SpiNNaker in 1 s real time. All results presented in this work ran reliably on SpiNNaker within this time scale.

The frequencies of the synthetic model inputs, both periodic and Poisson spike trains, are set to lie in the range 10–50 Hz at a resolution of 1 Hz. At each frequency, the model is simulated for 10 “trials,” each with a different seed, thus simulating multiple trials during SSVEP studies on humans. The output membrane potential time-series of all neurons in each population is averaged over time; this is done for all the 10 trials. The final membrane potential of each population in the model is the average of the mean membrane potentials across all the 10 trials. The “model output” is the membrane potential time-series of the TCR populations that are the main carriers of sensory information to the visual cortex.

**Figure 5:**
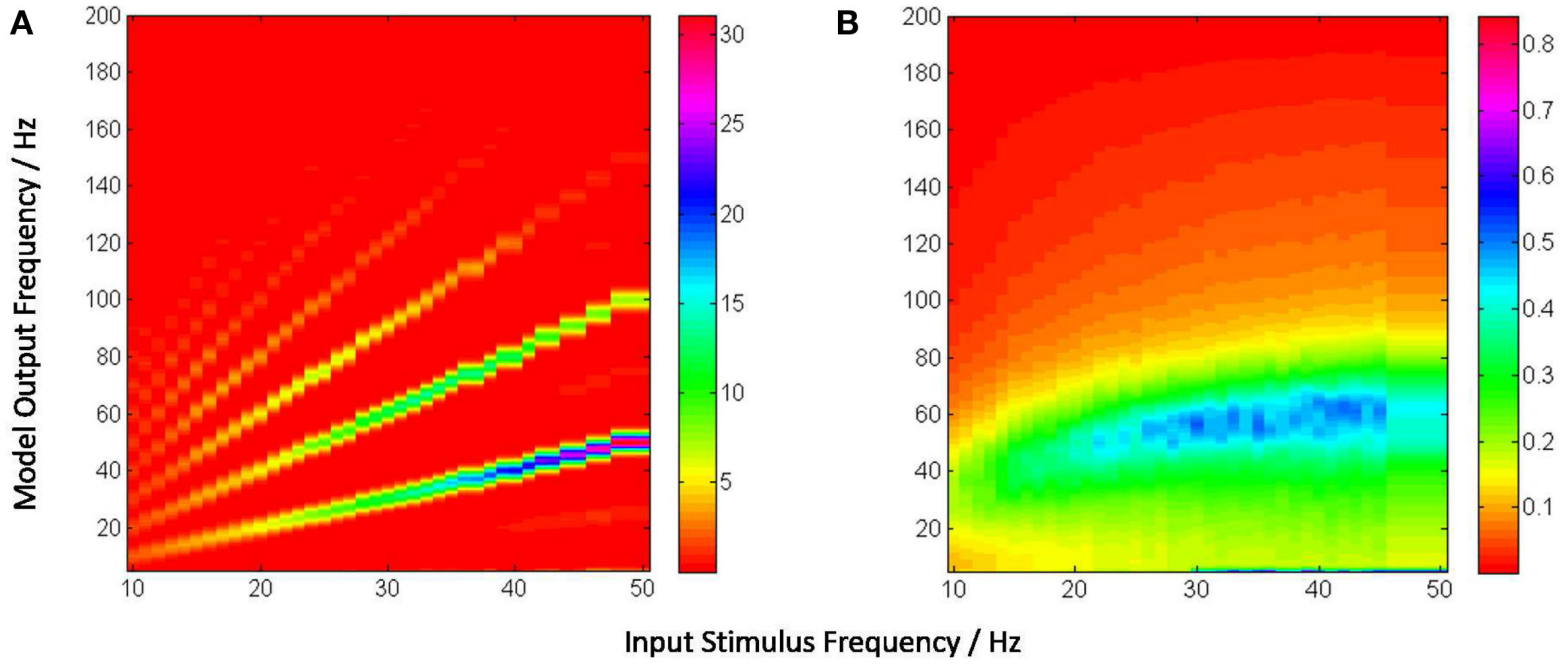
**(A)** Power spectra of the model output (y-axis) show a linear relation between the fundamental response frequencies and the periodic input at frequencies in the range 10–50 Hz at intervals of 1 Hz (x-axis). There is a prominent second harmonic of the fundamental frequency for all frequencies in the tested range. **(B)** Model power spectra with a Poisson (a-periodic) input spike-train at frequency ranges similar to as in **(A)**. The power spectra is stationary with maximum power within the range 40–60 Hz.

**Figure 6:**
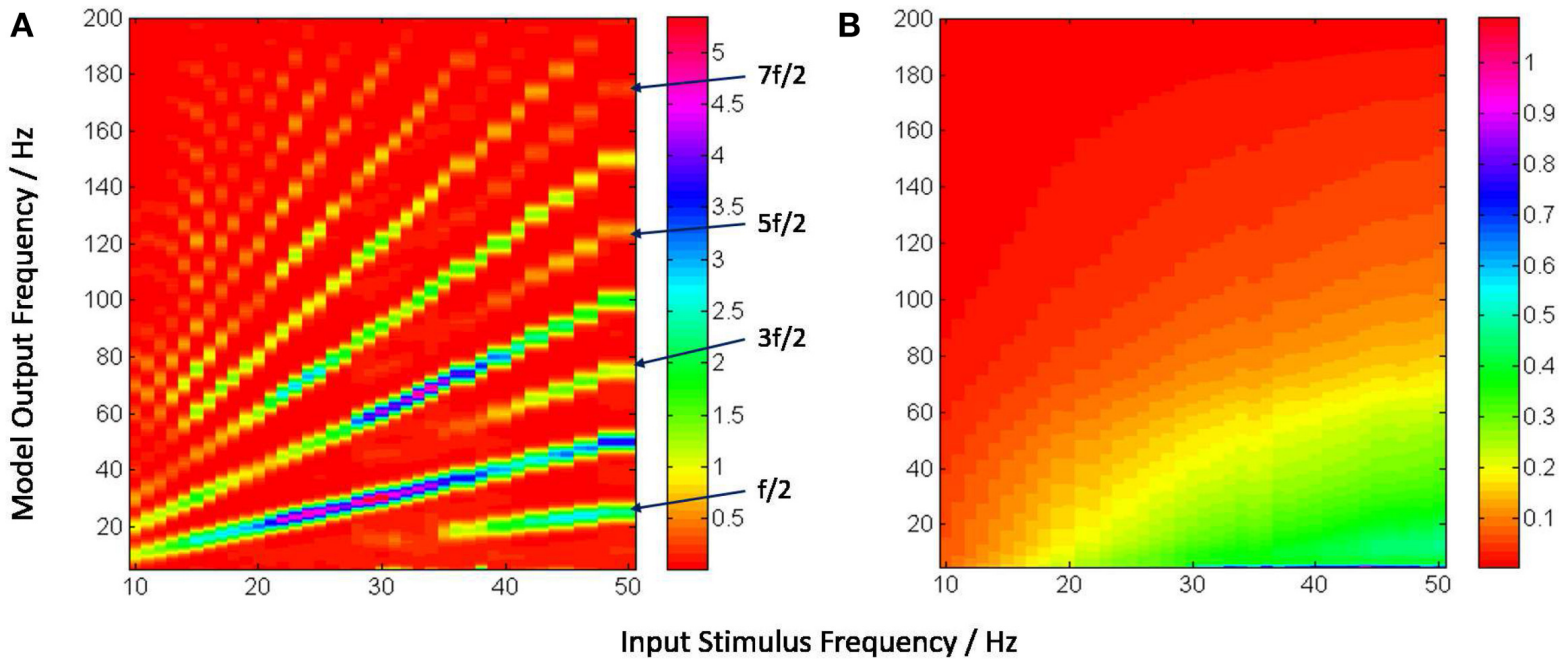
The model output power spectra (y-axis) corresponding to **(A)** periodic and **(B)** Poisson spike-train inputs in the range 10–50 Hz at a resolution of 1 Hz (x-axis), and spanning the upper-alpha (10–13 Hz), beta (14–30 Hz) and gamma (>30 Hz) EEG frequency bands. The figures correspond to the case when the IN feed-forward inhibition is suppressed in the circuit and the TRN feedback inhibition is dominant. The power spectra in **(A)** show distinct subharmonic contents for frequencies >40 Hz, and are indicated by arrows.

For frequency analysis, an epoch of 5 s from 0.5 s to 5.5 s of the average membrane potential time-series is down-sampled to 1,000 Hz (i.e., sampled at every 1 ms) and bandpass filtered between 1 and 100 Hz with a Butterworth filter of order 10. The filtered signal is then transformed using 4-point FFT and the power spectral density derived using the Welch periodogram. Images of the model output vs input stimulus frequency in Figures 5, 6 are generated using image processing toolbox commands in Matlab.

### 3.3. Entrainment of model output with periodic stimulus

Figure 5A depicts the model output power content against the input stimulus frequency, and is an attempt to compare with a similar classic depiction of experimental data corresponding to SSVEP in human EEG by Hermann (2001). However, Hermann's depiction was modified in subsequent research with the input stimuli frequencies (the independent variables) along the abscissa (Robinson et al., 2015). The latter approach is adopted by Labecki et al. (2016) as well as in this work as can be seen in Figures 5A, 6A. Our results indicate entrainment of the model output with the periodic visual stimulus and for all frequencies within the tested range of 10–50 Hz. Furthermore, harmonics at integral multiples of the fundamental frequency are also observed; 2nd and 3rd harmonic components are present for all frequencies, while upto the 5th harmonic component are observed for lower frequencies. Similar entrainment is also observed when the LGN model input is provided by the electronic retina output corresponding to periodic visual stimulus and is discussed in Section 3.5. In addition, the results imply a high fidelity performance of the circuit in response to periodic spike-train input across the upper-alpha (10–13 Hz), beta (14–30 Hz), and gamma (>30 Hz) frequency bands, and are in agreement with reports from experimental studies observed for occipital (Hermann, 2001; Notbohm et al., 2016) as well as parietal (Labecki et al., 2016) scalp electrodes in human EEG. However, there are no subharmonic components in the output as reported by both Labecki et al. (2016) and Hermann (2001).

When an aperiodic (Poisson) spike-train input is applied to the model, the evidence of entrainment of the output vanishes as shown in Figure 5B. The output power spectra show maximal power within the 40–60 Hz range corresponding to input frequencies ⪆20 Hz, implying an invariance to the change in the “parameter” (defining the spikes per second) of the Poisson distributed spike-train input. Also, the power amplitudes are significantly lower than those corresponding to the periodic inputs as indicated by the color bars.

### 3.4. Causality of interneurons on network dynamics

We compare the model output characteristics with the case when the IN has reduced inhibitory role in the LGN circuit, and the feed-back inhibition is dominant. This is simulated by decreasing the parameter values of the feed-forward synaptic connectivity from IN to the TCR population (*p*_*conn*_ = 0.07, *w*_*syn*_ = 1) with respect to their base values; simultaneously, the feed-back inhibitory synaptic connectivity parameters from the TRN to the TCR population are increased (*p*_*conn*_ = 0.309).

Figure 6A shows entrainment of the model output for all frequencies within the tested range of 10–50 Hz, and harmonics at integral multiples of the fundamental frequency *f*. Interestingly, subharmonic contents of the power spectra are now observed at $\frac{1}{2}f$ corresponding to the input frequency range of ≈ 36–50 Hz; for input frequencies ⪆40 Hz, sub-harmonic components are also observed at $\frac{3}{2}f$, $\frac{5}{2}f$ and $\frac{7}{2}f$.

The appearance of subharmonics in Figure 6A and their absence in Figure 5A imply a definitive role of the dominance of feedback inhibition in the system along with a suppression of the feed-forward inhibition. This validates the model-based results showing the non-linearity induced by the TRN feedback to the TCR in a simple mesoscopic-scale population model effects subharmonics in the model response (Labecki et al., 2016). However, the range of the input frequencies in our work are not in agreement with Hermann (2001) and Labecki et al. (2016), i.e., the model output do not show subharmonics for input frequencies within the alpha and beta bands (10–30 Hz).

In contrast, the power spectra corresponding to Poisson distributed spike-train input in Figure 6B shows a vertical shift in the dominant power “area” compared to Figure 6B, indicating an overall reduction in the dominant frequency of oscillation (“slowing” of power spectra) when the TRN feedback inhibition is dominant in the circuit; interestingly, this is in agreement with prior work on mesoscopic-scale population model (Sen-Bhattacharya et al., 2011a).

### 3.5. Model input from electronic retina: a comparative study

**Figure 7:**
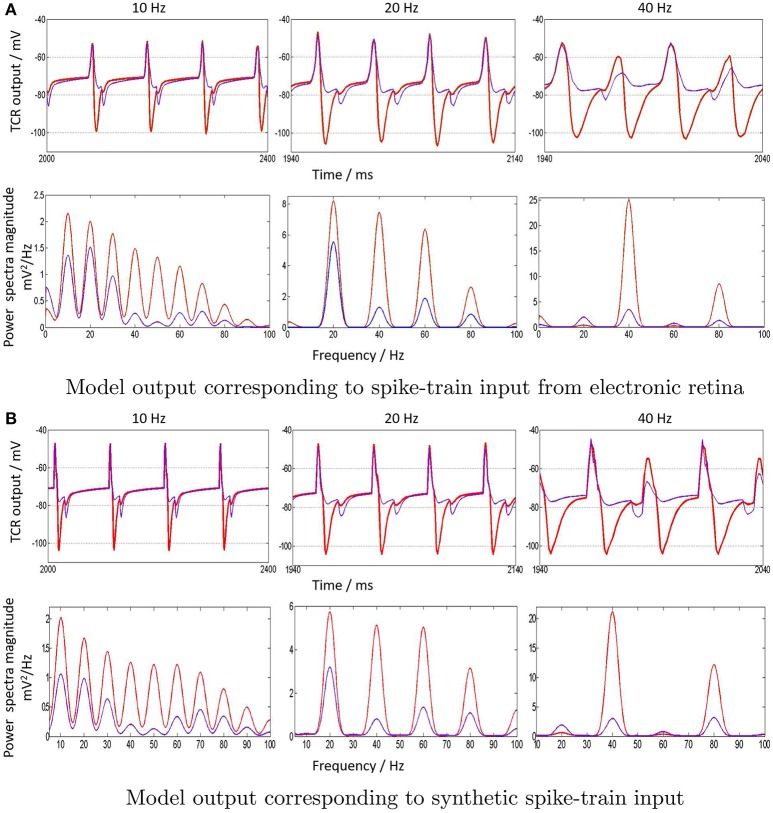
**(A)** “Zoomed-in” plots (red) of the model output time-series (top row) and corresponding power spectra (bottom row) in response to spike-train inputs generated by the electronic retina when subjected to periodic visual stimulation at 10, 20, and 40 Hz. The input frequencies are chosen as representative samples of upper-alpha (10–13 Hz), beta (14–30 Hz) and gamma (>30 Hz) EEG frequency bands that are associated with cognitive brain states. A comparative study, when the IN inhibition is suppressed in the LGN model with a simultaneous dominance of the TRN inhibition, is also indicated (blue) for all cases. **(B)** To validate the model output corresponding to synthetic periodic input stimulus generated in PyNN with the results in (a), the “zoomed in” time-series and power spectral density for 10, 20, and 40 Hz are shown. Both cases of when the IN is dominant (red) and suppressed (blue) are also displayed.

As elucidated in Section 2.3, retinal recordings that are made for the work presented here are at 10, 20, and 40 Hz periodic visual stimulus, and are representative samples of frequencies within the EEG upper-alpha, beta and gamma bands. The spike trains generated by the electronic retina for each of the three frequencies are provided as input to the LGN model. This is done for both cases when the IN plays the dominant inhibitory role within the LGN circuit, and when it is suppressed while feedback inhibition from TRN is dominant. Thus we have a total of six cases which are show-cased in Figure 7A. The top-panel in the figure shows “zoomed in” sections of the time-series such that four “cycles” of the model response can be observed. The entrainment of the model output is clear both from the time-series as well as the power density plots (bottom-panel).

The distinct effect of the IN inhibitory dominance in the LGN model is that the input signal periodicity is represented in the output with a high fidelity (shown in red). In contrast, when the TRN inhibition is dominant, this transmission efficacy is reduced and the output time-series (shown in blue) show characteristics of “half-wave” rectification, where the negative cycle is either clipped or minimally represented, and with a delay; the effect is more pronounced for higher frequencies i.e., 40 Hz. The corresponding power spectra reflect the reduced efficacy: the dominant frequency at the fundamental frequency *f* = 40 Hz is not distinct from its second harmonic content at 2*f* Hz, as well as the subharmonic at $\frac{f}{2}$ Hz. For 10 Hz, the dominant power is within the second harmonic at 20 Hz, and power within the harmonics >3*f* are significantly reduced compared to when the IN is dominant in the circuit. Thus, clearly, the model response loses the “frequency tagging” characteristics with a reduced effect of the IN feed-forward inhibition in the circuit corresponding to 10 and 40 Hz. Conversely, dominance of IN effects a true representation of the input frequency content in the circuit response.

The model outputs corresponding to synthetic periodic inputs in Figure 7B are in agreement with their respective counterparts corresponding to electronic retina input in Figure 7A. The only notable difference is at 10 Hz: the power spectra dominant frequency for 10 Hz retains the dominant characteristics of the input for the case when IN is suppressed in the circuit. Furthermore, the response to 20 Hz input is relatively unaffected by inhibitory role changes in the model, with only an amplitude reduction in both fundamental and harmonic power. This observation is consistent corresponding to both synthetic and electronic retina inputs and may suggest a resonant frequency of the circuit around 20 Hz; this may be investigated further in future work.

### 3.6. LGN multi-node architecture: studying alpha band behavior

**Figure 8:**
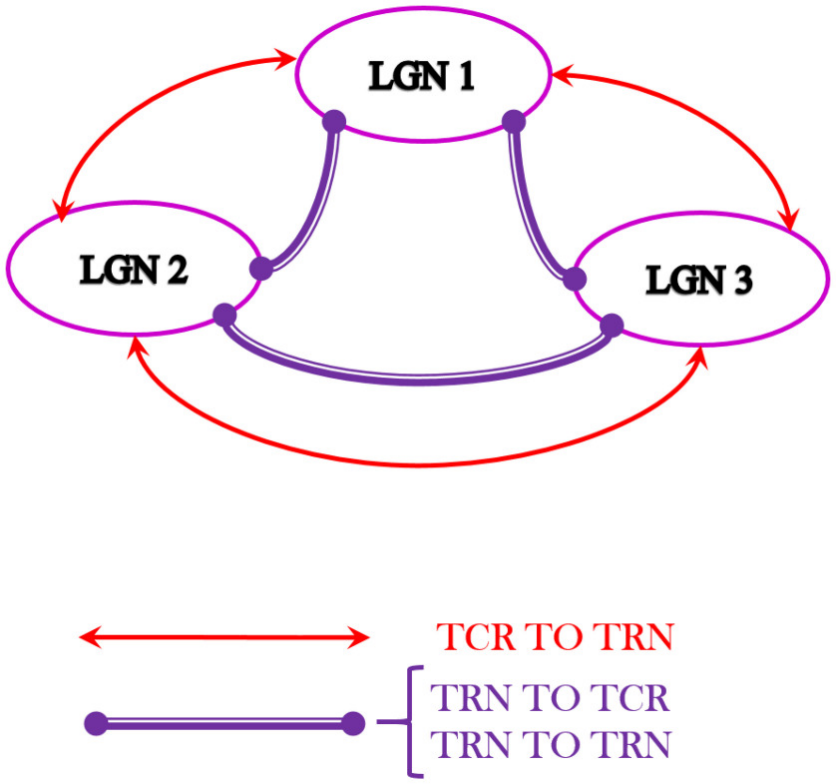
The multi-node LGN model synaptic layout with three instances of the LGN model shown in Figure 1, consisting of a total of 420 neurons. The TCR (TRN) cells of each node make excitatory (inhibitory) synapses to the TRN (TCR) cells of every other node. Also, the TRN cells of every node make inhibitory synapses to the TRN cells of every other node. For simplicity, the inter-node synaptic connectivity attributes *p*_*conn*_, *w*_*syn*_, *d*_*conn*_ are maintained as in respective pathways of the basic LGN model.

It is widely believed that the lower band rhythms in the EEG, for example the alpha rhythms, are a result of synchronous oscillations in a larger population of neurons; in comparison, higher frequency rhythms are known to involve smaller and localized neuronal groups (Buzsáki, 2006). To test the effect of an increased neuronal population corresponding to model input within the alpha band, we simulate a multi-node architecture of the LGN as shown in Figure 8.

Three “instances” of the LGN model shown in Figure 1 are spatially arranged (virtually) as three vertices of an equilateral triangle where all vertices are “nearest neighbors” of one another. Thus, inter-node connectivities in the network are simulated to be equidistant, and may be assumed to have similar time-scales of synaptic transmission. Interneurons are known to be inherently local and rarely project outside their respective local radii. Thus, the inter-node connectivities in the model are between the TCR and TRN populations and recurrent inhibition between the TRN populations. For example, the connectivity between *LGN 1* and *LGN 2* is thus: the TCR population of *LGN 1* (*LGN 2*) make excitatory connections on the TRN population of *LGN 2* (*LGN 1*); the TRN population of *LGN 1* (*LGN 2*) make inhibitory connections on both the TCR and the TRN populations of *LGN 2* (*LGN 1*); and so on. The resulting architecture in Figure 8 consists of 420 neurons.

The synaptic connectivity parameters and attributes for inter-node projections are maintained at similar values to those for intra-node projections for brevity. Along similar lines, the design consideration for three vertices in this work is to avoid complex calculations of synaptic delay for inter-node connectivities that are not nearest neighbors, unlike the simple case of our initial test framework in Figure 8.

**Figure 9:**
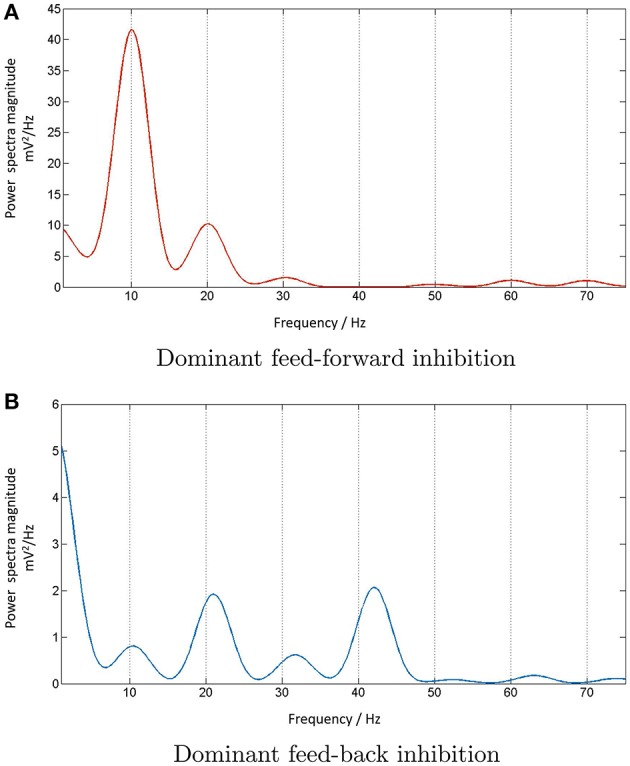
**(A)** The power spectral density plot corresponding to 10 Hz periodic input to the multi-node LGN model implies entrainment of the model response when the IN is dominant in the circuit. However, the output loses the fidelity when **(B)** the IN is suppressed in the circuit and the TRN inhibition is dominant. The dominant frequency is now at twice the fundamental frequency and reflects the frequency traits of the dominant TRN inhibition in the circuit (not shown).

A synthetic periodic spike-train input at 10 Hz generated in PyNN is now provided to all nodes of the model shown in Figure 8. The TCR membrane potential time-series from all nodes are collected separately at the end of model simulation on SpiNNaker. The mean of these three data sets provides a mean membrane potential, which represents the output of the multi-node model. The power spectral density plot of this multi-node model output is shown in Figure 9A. The power at the peak frequency of 10 Hz is now significantly higher than that within the second harmonic compared to the corresponding response of a single node of the model shown in Figure 7B. However, with dominant feedback inhibition from TRN, the model output loses its frequency tagging characteristics and is shown in Figure 9B. Such a response of the scaled up model may be compared to the “indifference” to IN suppression in Figure 7B (left panel), thus confirming our speculation on a larger neuronal tissue being involved in alpha band oscillations.

### 3.7. Evaluating power consumption

**Figure 10:**
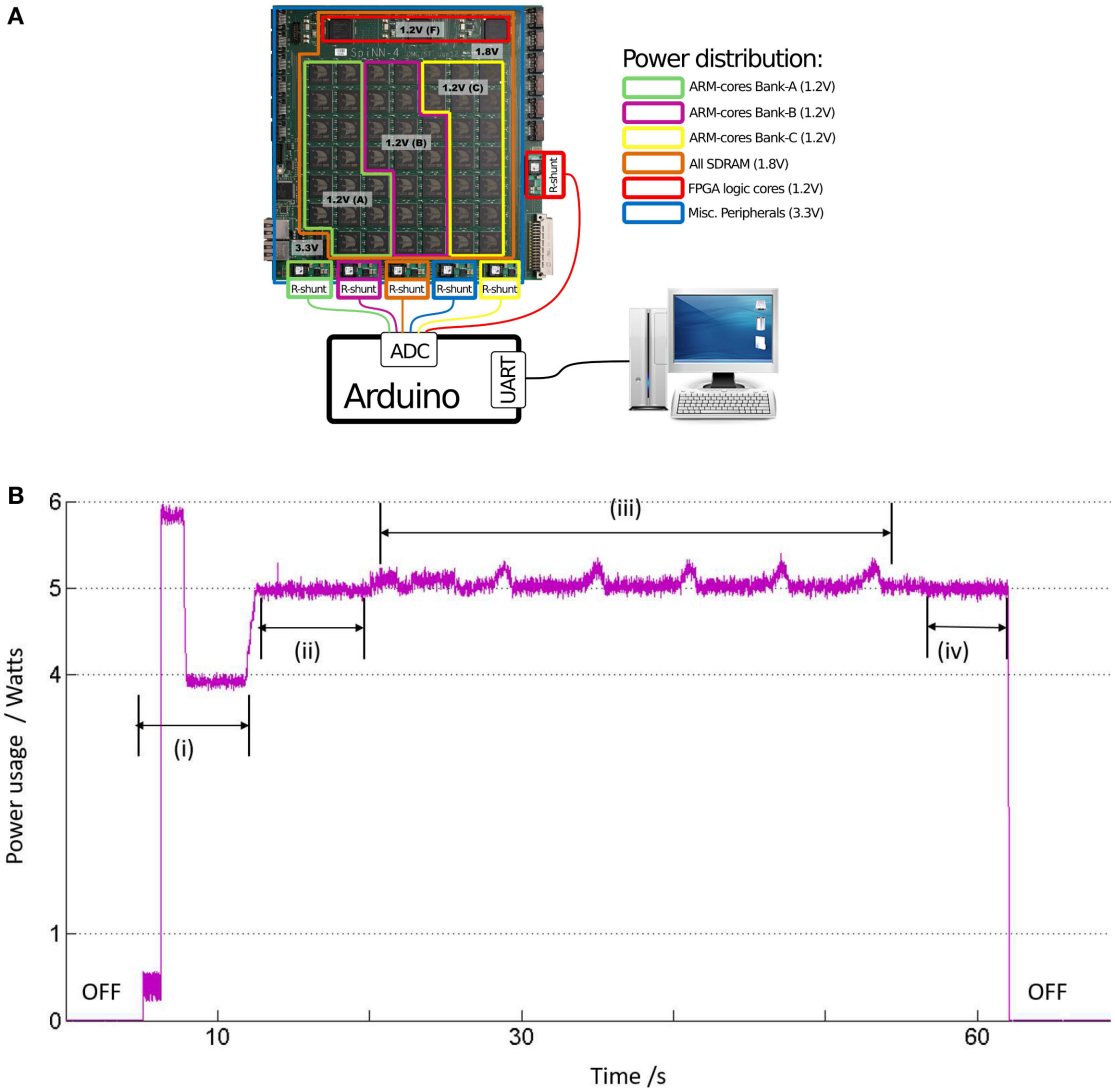
**(A)** The SpiNNaker machine as a 48-node board, where each “node” is the SpiNNaker chip. The chips are divided into 3 “ARM-cores bank” based on their common sources of power supply via a DC/DC converter, each of 1.2 V. The resistors referred to as “R-shunt” are used to measure the current drawn from power supply by the various board components. The power consumed in Watts is calculated by the Arduino board, which is then communicated to a normal desktop computer via a serial port. **(B)** The instantaneous power consumption by the LGN model running on one SpiNNaker chip in ARM-cores Bank-A: (i) When the machine is powered, the average power consumption settles down to ≈4 W after a short period of activity. (ii) When the machine is booted, the average power consumption by the core bank increases to an average of 5 W. (iii) With start of simulation, the instantaneous power rides a slow modulating wave with peak to peak power of around 300 mW, corresponding to work done during neural computation by the cores. (iv) After completion of simulation, a drop in average power to around 5 W indicate a return to the boot state.

A basic set-up that is developed in-house to estimate the power consumption by a board with 48 SpiNNaker chips (also referred to as 48-node board) is shown in Figure 10A. The SpiNNaker board has several components for board management and communication, which are powered by six DC/DC converters. Each converter has voltage rating sufficient for powering up specific blocks of components on the board: (a) 1.2 V for powering up a specific block of nodes, referred to as “ARM-core banks” in the legend of Figure 10A. The nodes on the board are organized into three such “ARM-core banks” labeled as “A–C” and powered by three of the DC/DC converters; (b) 1.8 V for SDRAM in all chips; (c) 1.2 V for FPGA logic cores; and (d) 3.3 V for several other components such as the Board Management Processor (BMP), the Ethernet (ETH) circuitry, indicator LEDS, etc. To measure the power delivered by each DC/DC converter, a resistor (“R-shunt” in Figure 10A) is placed in series with the converter. The current drawn through each R-shunt is converted into voltage and recorded by the Analogue-to-Digital (ADC) circuitry of an Arduino board. The Arduino calculates the power delivered by the DC/DC converters in Watts, and sends the result to a desktop computer for logging and visualization via a serial port.

A software report generated by sPyNNaker (available to the user upon completion of each simulation) indicates upto 8 processors being utilized by our LGN model, and on a single chip in ARM-cores Bank-A. Figure 10B shows the instantaneous power consumption by ARM-cores Bank-A. The board is switched on around after 5 s from the start of recording time. The total power consumed by all the ARM-cores Banks is around 12 W, and the power plots for ARM-cores Bank-B and C are same as that of ARM-cores Bank-A shown in Figure 10B, indicating an average consumption of 4 W per ARM-cores Bank. The power consumption due to other components in the circuit viz. FPGA, SDRAM, BMP, ETH-port, LEDs, and which are not affected by model simulation, is around 12 W. This agrees with the observed total power drawn during this idling condition (i.e., power switched on, but board not booted) *P* ≈ 24*W*. The machine is booted at around 12 s and the power plots for each ARM-cores Bank show a step increase of average power *P*_*boot*_ ≈ 5*W*. The corresponding total power *P* ≈ 27*W*. However, during the LGN model simulation from around 20 to 50 s, the power plot of ARM-cores Bank-A is observed to ride on a slow modulating wave at ≈0.16 Hz, and with a peak-to-peak amplitude of *P*_*sim*_ ≈ 300*mW*. As expected, the corresponding plots for ARM-cores Bank-B and C do not show any effects of the running simulation and stays stable at 5 W. The corresponding total power *P* ≈ 27–28 W.

Therefore, the overall power consumed by the model simulation on SpiNNaker *P*_*sim*_ ≪ 1*W*. This conforms with theoretical estimations that are based on prior research reporting ≈1 *W* power consumed per SpiNNaker chip working full load and simulating 256 neurons per ARM processor (each chip has 17 ARM processors available for neuronal model simulation) (Painkras et al., 2013). This is a rough indication of the power consumption for our model to be ≪1 *W*, as it uses only 8 ARM processors within a single chip (above-mentioned). It is worth mentioning here that the periodicity of the slow wave in Figure 10B is not an artefact of the periodic nature of the model input and is invariant to change in input spike train frequencies, as well as to aperiodic model stimulus. The exact source of this slow wave is currently being investigated.

## 4. Discussion

We have presented a spiking neural network model of the Lateral Geniculate Nucleus (LGN), the thalamic nucleus in the visual pathway, simulated on the SpiNNaker machine, which uses state-of-the-art low-power digital neuromorphic framework to facilitate massively parallel implementation of spiking neural networks in real time for time-steps *dt* ⩾ 1 ms. The synaptic layout of the model is consistent with biology and consists of three cell populations viz. the thalamo-cortical relay cells (TCR: the main carriers of visual sensory information to the cortex), thalamic interneurons (IN: the main source of feed-forward inhibition in the retino-geniculate pathway), and the thalamic reticular nucleus (TRN: a thin sheet of inhibitory tissue that is a major source of feedback inhibition for the TCR cells). The total number of neurons in the model is 140, representing an area of thalamic tissue spanning a few micrometers. The justification of designing and studying such a “tiny” (in biological terms) network on the SpiNNaker machine, which is a contradiction-in-terms with the design objectives of the latter, is to develop a robust and flexible “basic building block” that can be used for designing and developing larger modular frameworks on the SpiNNaker machine in future.

The motivation behind modeling the LGN has been to emulate real time biologically plausible dynamic visual processing using the SpiNNaker machine. One paradigm that is particularly gaining popularity in both clinical neuroscience as well a BCI research is the steady state visually evoked potentials (SSVEP), which are signals recorded via scalp electroencephalography (EEG) corresponding to periodic visual stimulus input. The “frequency tagging” (of the input stimuli) characteristics of the SSVEP signal facilitated by a high signal to noise ratio make it an “easy-to-read” tool for studying brain mechanisms in an awake cognitive state. It is worth mentioning here that the SSVEP (like EEG) signals are a representation of oscillations of the neocortical neuronal populations that lie beneath the recording scalp electrodes. On the other hand, recordings from the thalamus (TCR) cells are obtained largely via local field potentials (LFP). Thus, in principle, our model output mimics the LFP corresponding to periodic visual stimulus. Experimental research has shown that the LFP recorded from TCR cells of the LGN bear a strong correlation with EEG recorded over the occipital lobe (where the visual cortex resides) in a healthy brain. Moreover, it is now known that the thalamus is a key player in generating and sustaining brain rhythms observed via EEG. Based on such reports and findings, we validate our model results with the key findings of SSVEP recorded from human EEG by Hermann (2001), as well as with a combined experimental and model-based validation study by Labecki et al. (2016).

The model is simulated with synthetic periodic visual stimuli in the range 10–50 Hz at a resolution of 1 Hz. The model output validates experimental reports of entrainment with the periodic stimuli, displaying high fidelity in the fundamental frequency, as well as displaying harmonics of the fundamental frequency. However, there are no observed subharmonics of the fundamental frequency as reported by Hermann (2001). Indeed, the neuronal mechanisms that underpin harmonics and subharmonics in SSVEP are yet unclear and there is no consensus on their definitive source(s) (Notbohm et al., 2016). The authors in Labecki et al. (2016) demonstrate that even the non-linearities in a simple closed-loop circuit consisting of two cell populations can validate the SSVEP-like harmonics and subharmonics demonstrated by Hermann (2001). While the model used in this work indeed has such a closed-loop feedback circuit consisting of the TCR and TRN, also present is a feed-forward inhibitory circuit fed to the TCR by the LGN interneurons, which in turn receive direct input stimuli from the retinal spiking neurons. In a recent mesoscopic-scale population model-based study of the LGN dynamics, IN population feed-forward inhibition is implicated as a key attribute for ensuring efficacy of information transmission in the retino-geniculo-cortical pathway (Sen-Bhattacharya et al., 2016). This is aligned with experimental and theoretical studies emphasizing the key function of the IN in information processing corresponding to “attentive” brain states.

Along these lines, the IN synaptic pathway in the model is suppressed by changing appropriate synaptic connectivity parameters, at the same time the TRN feedback inhibition in the circuit is made dominant. The model output displays entrainment; additionally, the power spectra display subharmonic components for frequencies in the gamma (>30 Hz) range, which is associated with attentive and working brain states. The results indicate a distinct causality of the IN on the LGN dynamics, emphasizing the lead role of IN in maintaining information fidelity during cognitive processing. Furthermore, the results agree with the model-based study by Labecki et al. (2016), which did not consider any feed-forward inhibition, and thus simulated a condition similar to TRN dominant inhibition in the LGN model in this work. However, unlike in both these experimental (Hermann, 2001) and model-based (Labecki et al., 2016) reports, we did not note any subharmonic content in the model response corresponding to input frequencies within the tested upper-alpha (10–13 Hz) and beta band (14–30 Hz) frequencies.

To simulate real time periodic visual stimulus to the model, a state-of-the-art electronic retina, developed at the Instituto de Microelectronica de Sevilla, Spain, and based on a dynamic vision sensor technology, is used to record periodic ON/OFF stimuli from an LED in a controlled laboratory environment. The recordings made for the purposes of this work are at 10, 20, and 40 Hz, representative samples of the upper-alpha, beta and gamma band EEG frequencies, respectively. The model response validates experimental findings of entrainment with periodic input stimulus. Both cases corresponding to the IN being dominant or suppressed in the LGN circuit are tested with the electronic retina-generated spike trains. The power spectral plots corresponding to the 20 and 40 Hz inputs are in agreement with the model response corresponding to the synthetic input stimulus. As reported above, the 40 Hz power spectral plot shows subharmonic contents when the IN feed-forward inhibition is suppressed in the circuit; in addition, the distinct “frequency tagging” of the input frequency is lost for 40 Hz (but not for 20 Hz) and amplitudes of the fundamental, harmonic and subharmonic components are not significantly different.

In addition to the loss of fidelity with suppression of IN inhibition in the model output corresponding to the 40 Hz spike-train input from the electronic retina, we also note a similar loss in fidelity corresponding to the 10 Hz input from the electronic retina, when the maximum output power is within the second harmonic. However, this is not the case for the synthetic model input, and is unlike in a recent work with population models of the LGN (Sen-Bhattacharya et al., 2016), where a distinct causality is observed between the role of IN in the LGN and the circuit output within the alpha (8–13 Hz) band. One reason may be the noise in the electronic retina output spike-train, reflecting a realistic external environment that drives the retinal spiking neuron. In comparison, the model synthetic data is devoid of any noise. Another speculation toward the difference in behavior is the reduced number of neurons in our LGN model; lower frequency band EEG rhythms, for example alpha rhythms, are known to be generated by synchronous behavior of larger neuronal populations compared to localized population activity corresponding to higher frequencies. To test the hypothesis, we scale up our model architecture by creating three “instances” of the “basic building block” LGN model, assumed to be arranged spatially as three nodes of a lattice, which is a well known arrangement in the retinal cell space. Thus, we have simulated a relatively larger neuronal population of the LGN tissue consisting of 420 neurons. Indeed, the response of this multi-nodal network architecture corresponding to suppression of IN inhibition in the circuit shows a loss of output fidelity corresponding to a 10 Hz periodic visual stimulus. A more rigorous testing, and for frequency ranges wider than the current range, is suggested as future work on further scaled up multi-nodal LGN model.

To consolidate our observation of model entrainment with periodic input, and to confirm that such behavior is not an artefact of the materials and methods adopted in this work, we have tested the model with aperiodic visual stimulus. This is simulated by a spike-train following Poisson distribution with parameters in the same range as the periodic input frequencies. Results show no trace of entrainment. Furthermore, the model output power spectra is invariant in the tested range. However, this would need to be confirmed with larger LGN networks on the SpiNNaker, which is aligned to the short-term plans for future work. In contrast, our study shows that corresponding to periodic stimulus, even the small network is able to simulate SSVEP, which is a higher-level network dynamics. As above-mentioned, these observations will be further tested with a scaled-up LGN model as a part of ongoing research.

We note three specific areas that may contribute to enhance the framework presented here:

First, the power spectra and amplitude in the model are bound to change with increased stimulus strength, which can be effected in the model by changing connectivity parameter attributes. In the current model, these attributes are set to the minimal threshold values for initiating a spike output response in the model. During preliminary investigations, we have indeed noted increases in both the spike rate and amplitude of membrane voltage for increased connectivity parameter values, along the lines as noted in Notbohm et al. (2016). We leave this to be taken up in future research on the model.

Second, we have explored the tonic behavior for all cell types. However, it is known that the bursting nature for all cell types in the LGN underpins brain rhythms not only in the resting state but also in regulating attention. Simulating both tonic and bursting behavior in the LGN will certainly enhance the biological plausibility of the model.

Third, introducing synaptic plasticity in the model will enable implementation of learning rules, which will be vital toward introducing “intelligence” to the model in future works.

It may be noted that for brevity in this work, the retinal spike-train output is not provided to the model in real time. However, the interface of the electronic retina with the SpiNNaker machine is already available (Galluppi et al., 2012); thus, real time input using the interface can be potentially considered as a future work. Another point to note is that the SpiNNaker toolchain, sPyNNaker, provides access to several spiking neuron models other than the Izhikevich neuron models (“IZK-curr-exp”) used in this work. We have discussed above the modular structure of our LGN architecture; besides being easy to scale up, such a “basic building block” approach provides a “friendly” flexible substrate to explore the repertoire of spiking neuron model options provided by sPyNNaker.

The main drawback of the model is the lack of cortical circuitry, as thalamo-cortico-thalamic dynamics form the basis of brain rhythms observed via EEG and LFP. However, a decorticated (disconnected from the cortex) model of the LGN in this work is by design rather than any other constraint, and is justified as a necessary step prior to building larger interfaced structures; similarities can be drawn with several decades of research on isolated thalamic slices of mammals and rodents, that has paved the way for the current advanced understanding of the thalamo-cortical dynamics.

In terms of performance evaluation of model simulation on SpiNNaker, two areas take precedence: Real time implementation—we note that the differential equations defining a single neuro-computational unit in the model (current-based Izhikevich's neuron) need a 0.1 ms time-step for solution accuracy. However, the SpiNNaker hardware is designed inherently to simulate at time-steps ⩾1 ms in real time. Thus, all simulation of the LGN model on SpiNNaker ran 10 times slower than real time; for example, if the simulation duration is set to 3 s with a time-step of 0.1 ms in the PyNN code describing the LGN model, the actual execution time of the model on SpiNNaker is 30 s with a time-step of 1 ms in real time. However, such scaling up of simulation time is not a concern for this small LGN network, serving as a test-bed for future large-scale simulations, particularly because the model is guaranteed to run in the expected time. However, sPyNNaker is in development mode, and we do expect to see issues upon scaling up the model. Thus, we expect to be able to provide a more realistic evaluation on the real time performance of SpiNNaker when we run scaled-up versions of this model, as well as others that are under development.

Power consumption—Prior work has provided estimated figures for maximum power consumption by a SpiNNaker chip as 1 W. Simulation of the LGN network uses 8 (of 17 available) processors on a single SpiNNaker chip, implying a power consumption ≪1 *W*. This is verified by an in-house power measurement set-up that records the active power (wattage) drawn from the mains by the SpiNNaker board. This demonstrates a very low power consumption by our LGN model on the SpiNNaker platform.

## 5. Conclusion

Computational neurology and psychiatry provide an excellent means for in-depth investigations of vital structures such as the thalamus, which are otherwise hard to study in wet-laboratories (Sen-Bhattacharya et al., 2017). Besides dealing with sensory and cortical inputs, the thalamic nuclei play strategic roles in the functioning of the limbic brain, and are known to link decision making and action selection circuitry to the motor circuitry. Thalamic dysfunction is often believed to underpin several neurological and psychiatric disorders. On the other hand, the thalamus forms the primary output target of the Basal Ganglia (BG) circuit, a brain structure and mechanism that has been the primary focus of the autonomous robotics community toward incorporating learning and decision making in machines (Humphries and Gurney, 2002). Advanced frameworks for building large-scale biologically plausible models such as the SpiNNaker provide a timely impetus to computational model-based research in neuroscience, and may inspire further research for understanding the functioning of the thalamus, a vital “cog” in the “wheel” that is the central nervous system. We believe that the spiking neural network model presented in this work, simulated on the SpiNNaker machine, and tested with realistic periodic stimulus from an electronic retina, will act as a “basic building block” toward future endeavors in both computational neuroscience and autonomous robotics.

## Author contributions

BS planned and designed the research and ran model simulation for generating results. TS performed data acquisition, data processing, model simulation and generating results related to electron retina. LB and AB made voluntary research contributions to the software code of the model; LB contributed toward object oriented Python script for the multi-node LGN architecture and related data visualization; AB contributed toward Python script for data processing and visualization of results. AR and AS supported with SpiNNaker toolchain and implementation of the model on SpiNNaker. IS performed power analysis of the model simulation on SpiNNaker. SF provided guidance and support on the research. BS, TS, AB, and IS generated the figures. BS, TS, AS, AR, IS, and SF wrote the manuscript.

### Conflict of interest statement

The authors declare that the research was conducted in the absence of any commercial or financial relationships that could be construed as a potential conflict of interest. The reviewer VV and handling Editor declared their shared affiliation, and the handling Editor states that the process nevertheless met the standards of a fair and objective review.
